# Preparation of Breathable Cellulose Based Polymeric Membranes with Enhanced Water Resistance for the Building Industry

**DOI:** 10.3390/ma14154310

**Published:** 2021-08-01

**Authors:** Atif Hussain, Pierre Blanchet

**Affiliations:** Department of Wood and Forest Sciences, Université Laval, Quebec City, QC G1V 0A6, Canada; pierre.blanchet@sbf.ulaval.ca

**Keywords:** cellulose fiber, vapor permeability, surface treatment, surfactant, bio-based materials

## Abstract

This study focuses on the development of advanced water-resistant bio-based membranes with enhanced vapour permeability for use within building envelopes. Building walls are vulnerable to moisture damage and mold growth due to water penetration, built-in moisture, and interstitial condensation. In this work, breathable composite membranes were prepared using micro-fibrillated cellulose fiber (CF) and polylactic acid (PLA). The chemical composition and physical structure of CF is responsible for its hydrophilic nature, which affects its compatibility with polymers and consequently its performance in the presence of excessive moisture conditions. To enhance the dispersibility of CF in the PLA polymer, the fibers were treated with an organic phosphoric acid ester-based surfactant. The hygroscopic properties of the PLA-CF composites were improved after surfactant treatment and the membranes were resistant to water yet permeable to vapor. Morphological examination of the surface showed better interfacial adhesion and enhanced dispersion of CF in the PLA matrix. Thermal analysis revealed that the surfactant treatment of CF enhanced the glass transition temperature and thermal stability of the composite samples. These bio-based membranes have immense potential as durable, eco-friendly, weather resistant barriers for the building industry as they can adapt to varying humidity conditions, thus allowing entrapped water vapor to pass through and escape the building, eventually prolonging the building life.

## 1. Introduction

The accumulation of water in the form of excessive moisture within building envelopes can lead to the premature deterioration of building materials [1]. Buildings can be affected by three main sources of moisture: external moisture from precipitation or groundwater; internal moisture from occupant presence and their activities; and built-in moisture in the materials accumulated during manufacturing. Moisture movement in building wall cavities is facilitated by air currents, heat transfer and diffusion through materials. The penetration and accumulation of excessive moisture in building envelopes can lead to interstitial condensation, which can cause structural damages, mould growth, as well as damage to indoor materials. The moisture flow in building envelopes can be controlled by using barriers in walls, floors, ceiling, and roofs, thereby preventing interstitial condensation [2].

Weather barriers are membranes used in the exterior side of the wall system and act like a shell for buildings [3]. A premium, high-performance weather barrier has four beneficial and essential functions: air resistance, water resistance, durability during construction, and the right level of vapor permeability. Although the process of vapour permeability is least understood and heavily ignored, it can greatly affect the wall performance [4]. Vapor permeability is also discussed in terms of breathability of the material as its ability to allow moisture or water vapour to pass through it [5,6]. A good weather barrier is expected to resist bulk water (in liquid form) but should not necessarily block water vapour (in gas form) [7]. In the past, installing barriers in envelopes was not necessary as the walls had very low insulation. However, in the current scenario when the wall interior gets wet, tighter enclosures combined with high levels of thermal insulation can significantly reduce the drying potential of the wall [8]. Vapor barriers have been used with the intention to protect walls from becoming wet, however their main disadvantage is that the barrier also prevents from drying if the interior of the walls get wet [9]. Therefore, there is a need to develop a barrier with a unique structure that can specifically allow vapour to pass through but resist water entering the building wall. The most common barriers currently used in industry are non-biodegradable fossil fuel-based, which also raises the need to develop new barriers with more sustainable properties.

Bio-based materials have recently become popular in the building and construction industry due to their insulation and hygroscopic properties [10,11]. Studies have reported that using these materials in construction increases the energy efficiency of the building and provide a comfortable and healthy indoor environment [12,13,14]. Some bio-based materials also have the ability to absorb and release moisture with respect to changing relative humidity levels, which can reduce the load on air conditioning and have a positive impact on wellbeing of residents [15,16]. The ability of bio-based materials to capture and lock CO_2_ from the atmosphere during their lifetime can be highly beneficial for the environment [17]. The Quebec building code highlights that greenhouse gas emissions from buildings account for a large share of the region’s overall emissions and strives to achieve its emission reduction targets by 2030 [18]. To accomplish this goal, buildings should have a highly energy efficient design as well as lower embodied energy. Since majority of the electricity generated for use in Quebec buildings is derived from hydroelectricity, which is a renewable source, the embodied energy of buildings plays a major role in contributing towards carbon emissions during their life cycle [19]. Quebec has immense potential for developing new renewable materials from wood by-products that will result in the production extremely lower embodied energy materials.

Cellulose is the most abundant organic compound, and it can be obtained quite easily from wood pulp in the form of very thin and long fibers. Recent advancements in science have supported the development of industrial processes for the extraction of cellulose fiber from wood in large-scale volumes, having a 100% yield without the use of enzymes or chemicals [20]. The fibres have unique properties such as high specific surface area, good strength and rheological properties making them a highly versatile, biodegradable, and compostable additive for a composite as either a membrane or a coating. Recent studies have used cellulose fibres for the development of films addressing mechanical [21,22] and optical properties [23,24]. However, a few studies have reported excellent vapor barrier properties in packaging applications [25,26,27] and their use in the construction industry [28]. 

Cellulose is highly hydrophilic due to the presence of hydroxyls in its chemical structure. High moisture sensitivity in bio-based materials can lead to fungal growth and compromise the durability of the material [29]. Furthermore, the quality of the end product can be affected during the manufacturing stages if cellulosic materials encounter humid environments or unexpected water [30]. The high water absorption capacity of bio-based materials also makes them incompatible with hydrophobic polymers causing poor interfacial adhesion in the composites [31,32]. Numerous studies have investigated the effect of alkali [33], acetylation [34], ionic liquids and salts [35,36,37], silane [38], sol-gel [39], and surfactant [40,41] treatment of bio-based materials that improve their hydrophobicity and compatibility with polymers.

Cellulose fibers offer wide possibilities for new product development and the objective of this research is the utilization of commercially available cellulose fibers with a bio-based polymer for the development of a breathable vapor barrier for the construction industry. The work also involves the treatment of the cellulose fibers and an investigation of their compatibility with the polymer to determine the vapor permeability, morphology, as well as physical and thermal characteristics of the composite material.

## 2. Materials and Methods

Cellulose fibers commercially referred as cellulose filaments were received from Kruger Biomaterials Inc. (Montreal, QC, Canada), a privately held company that transforms renewable resources into sustainable essentials. The cellulose fibers used in this study were obtained in the form of suspension (2.5 wt%). The fibers were obtained by freeze-drying the suspension. PLA grade 4043D was used in this study and obtained from NatureWorks (Minnetonka, MN, USA). This particular grade of PLA is targeted for the preparation of membranes and films. Chloroform was used as the solvent for dissolving PLA and obtained from Sigma-Aldrich (Oakville, ON, Canada).

### 2.1. Fiber Treatment

For the treatment of cellulose fibers, an organic phosphoric acid ester-based surfactant was added to the CF suspension. The surfactant was obtained from EMCO-Inortech (Shwego-wett 6267), Montreal, QC, Canada. It is a biodegradable, VOC-free, anionic universal wetting, and dispersive additive. The concentration of surfactant used for treatment of the fibers was 20 wt% of dry fiber weight. The fiber suspension along with the surfactant was mixed using a high shear mixer at 2000 rpm for 15 min and then freeze-dried.

### 2.2. Film Preparation

**Figure 1:**
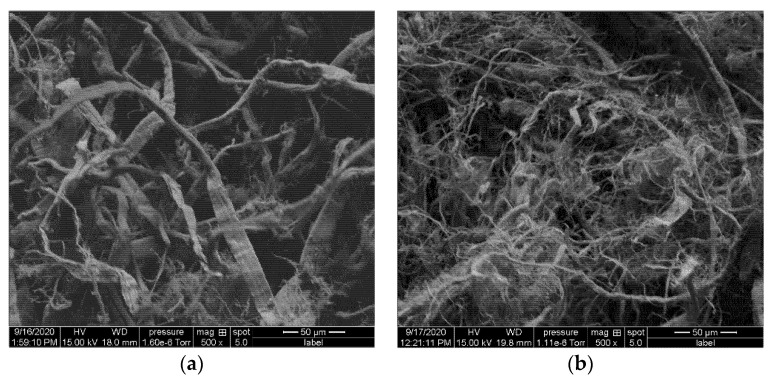
Micrographs of cellulose fibers (**a**) untreated, (**b**) after surfactant treatment.

**Figure 2:**
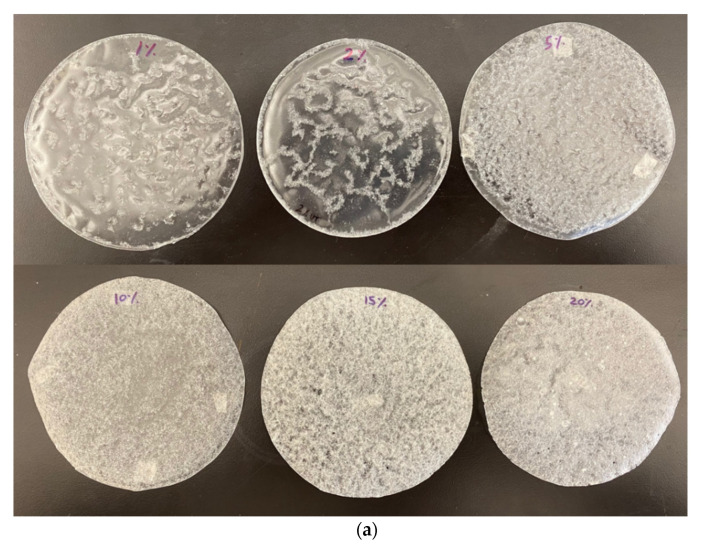

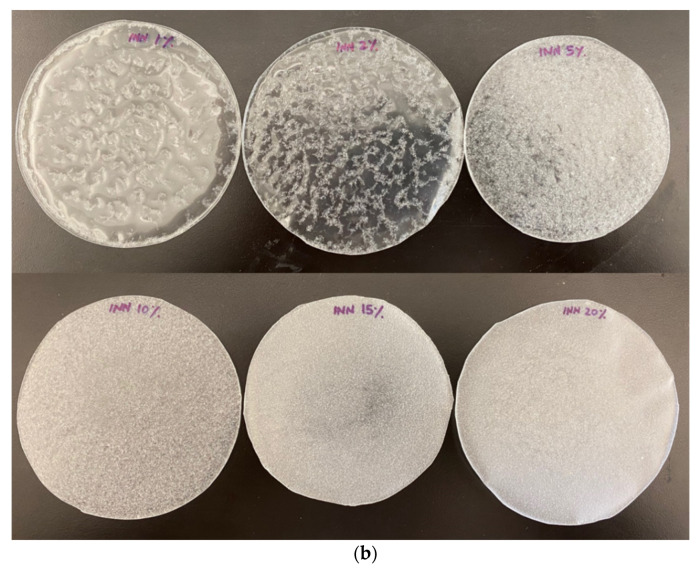
Photographs of (**a**) PLA-UCF films with 1–20% UCF content; (**b**) PLA-TCF films with 1–20% TCF content.

PLA membranes were prepared using solvent casting method. Briefly, 5 g of PLA was dissolved in 100 mL of chloroform under constant stirring at 600 rpm for 2 h at room temperature and atmospheric pressure. The solution was poured into a Petri dish and left in a fume hood at room temperature for evaporating the solvent. For preparation of the CF composite membranes, varying concentrations of untreated and surfactant treated CF were the added to dissolved PLA and stirred vigorously for another 30 min using a high shear mixer at 1200 rpm. At the end of the mixing process, the PLA-CF solution was homogenous, and no bubbles were observed. The prepared PLA membranes with varying concentrations of untreated cellulose fibers (UCF) and treated cellulose fibers (TCF) used for this study are mentioned in Table 1. Micrographs showing UCF and TCF are presented in Figure 1. Photographs of all prepared films used in this experiment are shown in Figure 2. All membrane samples were vacuum dried at 40 °C for 96 h to release any remaining solvent and then sealed and stored in zipped airtight bags for characterization.

### 2.3. Methods

#### 2.3.1. Vapor Permeability and Water Vapor Transmission Rate (WVTR)

The ability of a porous material to transfer moisture due to a vapour pressure gradient can be expressed by the vapour permeability of the material. The transfer of moisture during this process can take place due to three factors: diffusion (self-collision of water molecules), effusion (collision of water molecules with the pore walls), and liquid transfer (associated with capillary condensation) [42].

The vapor permeability and the WVTR of the composite membranes were determined according to ASTM standard E 96 using the wet cup method. The data used in this analysis were obtained a dynamic vapour sorption equipment DVS Advantage, SMS, London, UK. A SMS Payne Type Diffusion cell was used in this test that is specially designed to measure the permeability and moisture transmission rate of thin films in the DVS instrument. The Payne cell has two main components: cell lid that holds the test sample and a cell cup with a small reservoir. The test samples were cut to a diameter of 15.5–16.0 mm to perfectly fit in the Payne cell lid. The Payne cell cup was filled with 200 µL of water and the sample was placed between two O-rings. The bottom cell cup was screwed into the upper cell lid and placed on a metal sample pan for measurement in the DVS instrument. The samples were initially conditioned for 30 min at 95% RH and 25 °C. The test was run under 50% RH and 25 °C for 24 h and the cell was auto-weighed every minute by a DVS high mass ultra balance. The cell opening provided an active area of 113 mm^2^ for moisture transport. The sample thickness was measured using a micrometer and varied between the specimens (100–500 µm). The tests were performed in triplicate and the average reading was reported.

The WVTR was determined by dividing the slope of the linear portion of the weight gain versus time curve by the tested surface area (interior cell area = 113 mm^2^) of the sample using the following equation:

(1) $$\mathrm{WVTR}\;\left(\frac{g}{h\cdot m^{2}}\right)=\frac{\mathrm{slope}\;(g/h)}{\mathrm{cell}\;\mathrm{area}\;\left(m^{2}\right)}$$

WVTR describes the rate of water permeating through a test specimen into the headspace volume of a container, which differs in relative humidity (ΔRH).

Vapor permeability, P hence is represented as:

(2) $$P\;\left(\frac{g}{h\cdot m\cdot \mathrm{Pa}}\right)=\frac{\mathrm{WVTR}\;(g/h\cdot m^{2})\cdot d\;\left(m\right)}{\Delta P\;\left(\mathrm{Pa}\right)\;\left(m^{2}\right)}$$

where d is the test specimen thickness (m)

It is simpler to compare WVTR between samples with varying thicknesses, by eliminating the thickness factor and reporting it as:

(3) $$\mathrm{Normalized}\;\mathrm{WVTR}\;\left(\frac{g}{h\cdot m}\right)=\;\mathrm{WVTR}\;\left(\frac{g}{h\cdot m^{2}}\right)\cdot d\;\left(m\right)$$

#### 2.3.2. Water Retention

The water retention test was performed according to ASTM D-570-95 using the 24 h immersion test method. The specimen was placed in a container of water at room temperature, rested at its edge and entirely immersed. At the end of 24 h, the specimen was removed from water, wiped free of surface moisture with a dry cloth, and weighed to the nearest 0.001 g immediately. The test was performed in triplicates and the average reading was reported. The test specimens were thin films of 150 mm in diameter. 

The percentage of water absorption (WA) was calculated according to the following equation:

(4) $$\mathrm{WA}\;=\frac{m_{24}-{\;m}_{0}}{m_{0}}\times 100$$

where m_0_ is the initial mass of the test specimen (g), m_24_ is the mass of the test specimen after partial immersion for 24 h (g).

#### 2.3.3. Scanning Electron Microscopy

Photomicrographs of the samples were captured using a Thermo Fisher Scientific Scanning Electron Microscope (SEM), model FEI Quanta 250 (Hillsboro, OR, USA) operating at 15 kV. The samples were gold coated to obtain high magnification of morphology and texture.

#### 2.3.4. Thermogravimetric Analysis

The thermal degradation behaviour of samples was studied using a thermogravimetric analyser TGA/DTA 851e Mettler Toledo instrument (Columbus, OH, USA). Approximately 8–10 mg of sample was placed in an uncovered 70 µL alumina crucible to determine mass loss during heating. The specimens were heated at a rate of 10 °C/min from 25 to 950 °C under nitrogen atmosphere purged at 50 mL/min. The test was performed in triplicate.

#### 2.3.5. Differential Scanning Calorimetry

The melting point and enthalpy of the samples were determined by a differential scanning calorimeter DSC Mettler Toledo 822/e (Columbus, OH, USA). Approximately 7 mg of sample was placed in a sealed aluminium cell to determine melting and glass transition temperatures and enthalpies during heating. The samples were heated from 25 °C to 250 °C at 10 °C/min under nitrogen. The test was performed in triplicate.

#### 2.3.6. Dynamic Mechanical Analysis

Glass transition temperature (Tg) was determined by dynamic mechanical analysis based on the maximum of the tan delta peak, which represents the ratio of storage modulus to loss modulus. The tests were conducted using a Q800 dynamic mechanical analyzer (DMA) from TA instruments, New Castle, DE, USA. The tests were conducted in tension mode at a frequency of 1 Hz and an amplitude of 10 µm. The test samples were cut from the films using a laser machine having a dimension of 20 mm (length) by 4.5 mm (wide) and approximate thickness of 0.2 mm. All specimens were initially conditioned at 25 °C in the DMA chamber, and then dynamic heating scans were performed from 25 to 150 °C at 3 °C/min. The test was performed in triplicate and the average reading was reported.

## 3. Results and Discussion

**Figure 3:**
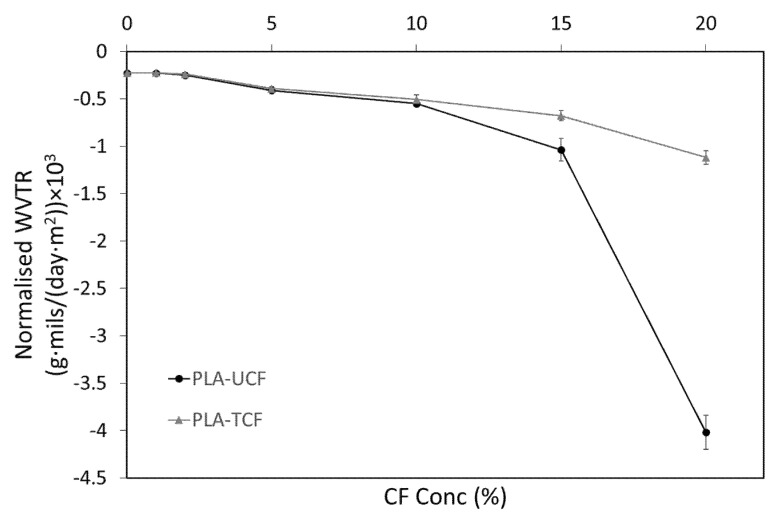
WVTR of the films with varying CF concentration.

Moisture transfer in porous materials takes place due to the presence of a vapour pressure gradient between their top and bottom surfaces. Figure 3 represents the water vapour transmission rate (WVTR) of the prepared membranes. It was seen that, overall, the vapour transmission increased with increasing CF content in the samples. The detailed data analysis of vapour permeability of the composites is reported in Table 2. 

The normalized WVTR is an important factor to consider when measuring permeability of composites with different thicknesses. The WVTR of the PLA-TCF was similar to PLA-UCF composites at lower CF concentrations. As the CF concentration increased beyond 10%, the composites with treated fibers showed significantly lower vapor transmission rates. The WVTR of PLA-TCF20 was 73% lower than that of PLA-UCF20. The increase in vapour permeability and WVTR at higher CF concentration is linked to the pore network and structure of the composite. The surfactant treatment resulted in good dispersion of fibers in the PLA matrix, thereby reducing formation of voids and limiting capillary movement of water molecules. The treatment, however, did not completely block microscopic pores in the samples that are needed for vapour diffusion.

The increase in the water vapour permeability at higher humidity level is related to the enhanced transport of moisture. This phenomenon is induced by the transfer of liquid in the microscopic pores of the material that are filled with water due to capillary condensation [42]. For materials that show hysteresis in their sorption isotherm, it has been reported earlier that their water vapour permeability is dependent on the moisture content [43].

**Figure 4:**
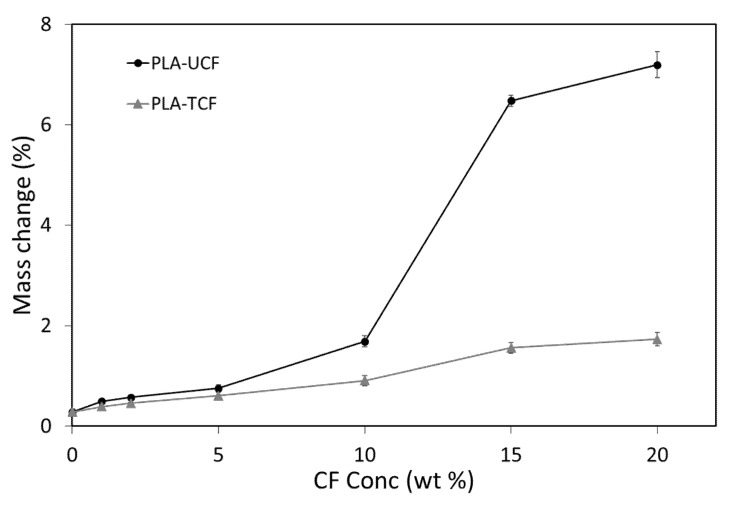
Water absorption behaviour of the films with varying CF concentration.

The water absorption of the composites was calculated as percentage of absorption with respect to initial mass. As seen in Figure 4, the water absorption behaviour of the composites was significantly affected by the treatment of CF keeping the WA values to a minimum. PLA-UCF composites showed higher water absorption due to the absence of treatment on CF. PLA-UCF20 showed the highest absorption having a WA value of over 7% for 24 h immersion.

When compared to the treated fiber composite having the same CF concentration (PLA-TCF20), PLA-UCF20 showed a four-fold increase in water absorption. In addition to enhanced dispersion due to the CF treatment, it is evident that the chemical structure of the CF has also been altered, reducing available surface hydroxyl groups that are responsible for the higher water uptake in the samples.

**Figure 5:**
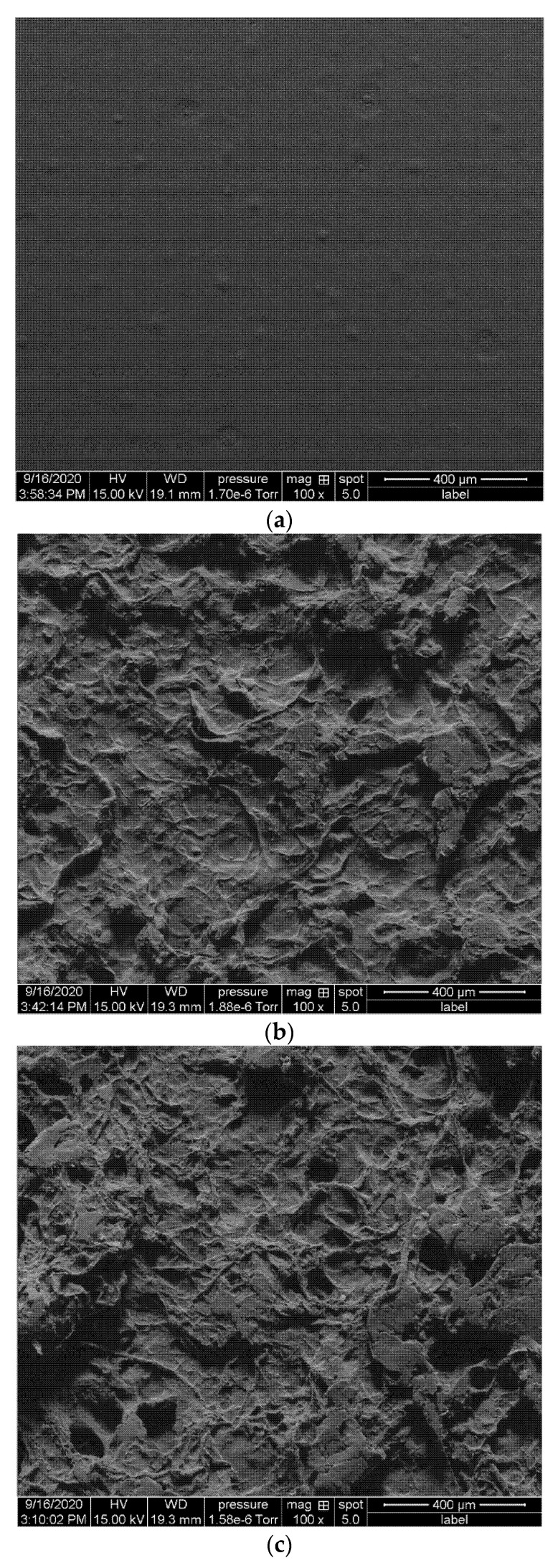

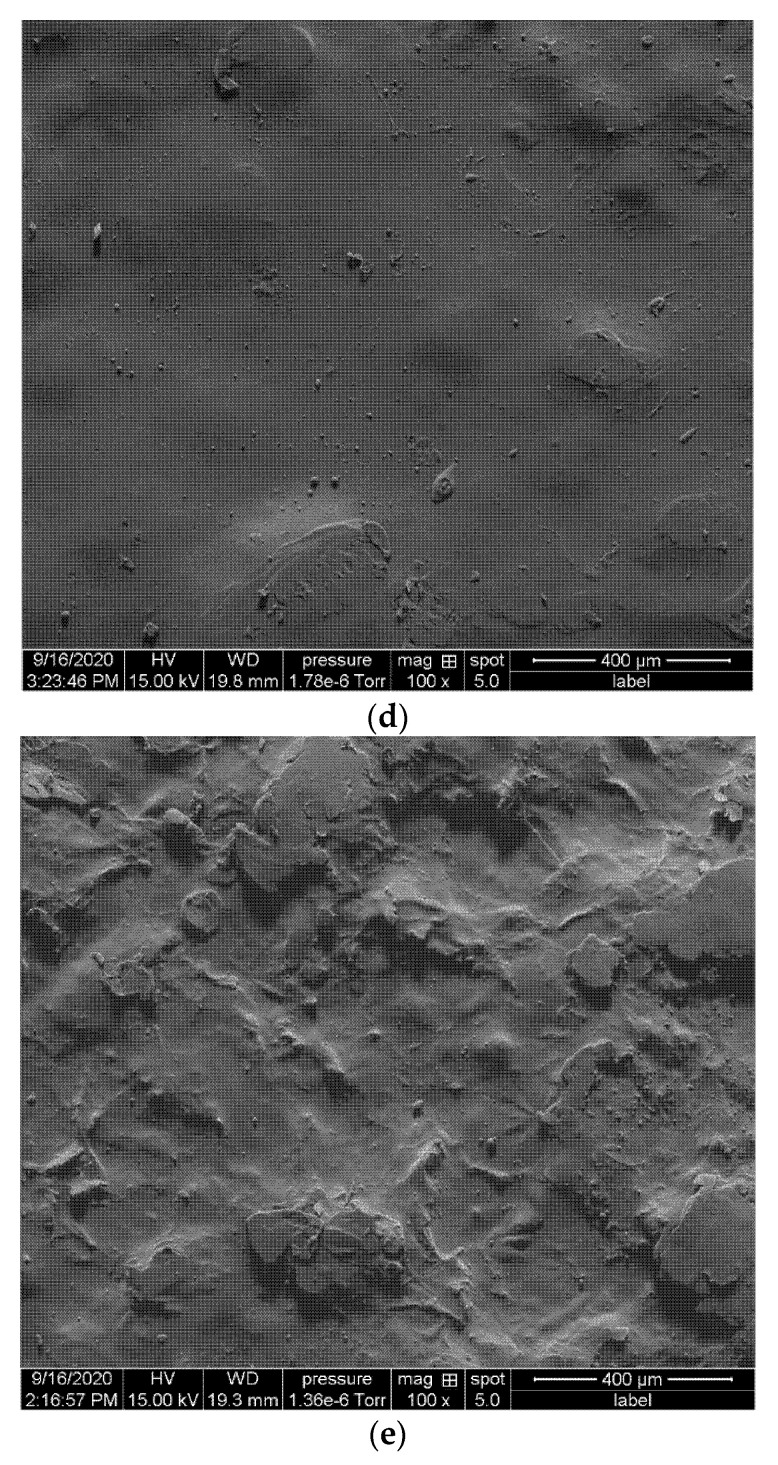
Micrograph of prepared films (**a**) PLA; (**b**) PLA-UCF10; (**c**) PLA-UCF20; (**d**) PLA-TCF10 and; (**e**) PLA-TCF20.

A close examination of the composite morphology revealed that samples without CF treatment showed poor dispersion in the PLA matrix whereas the treatment was found to be effective for preparing homogenous PLA-CF samples. In Figure 5b,c, it can be seen that the untreated fibers and PLA have poor interfacial adhesion and the fibers show agglomeration at the surface. On the other hand, Figure 5d,e show that samples with surfactant treated CF are well dispersed in the PLA matrix. 

The presence of the acid phosphate ester surfactant on the surface of nanocellulose facilitates their dispersion in the polymer matrix and improves the nucleation effect [40,44,45]. The surfactant acts as a stabilizing agent for obtaining a stable dispersion of cellulose fibers in the matrix [46,47]. The surfactant treatment has also been found to be effective in preventing the agglomeration of CF in solvents such as chloroform, thereby enhancing dispersibility in the polymer matrix [48].

The thermal properties of the PLA-CF films were investigated by TGA, DSC, and DMA analysis to determine the effect of surfactant treatment on the thermal stability of the polymer composites. The thermal characteristics of the composites are mentioned in Table 3.

**Figure 6:**
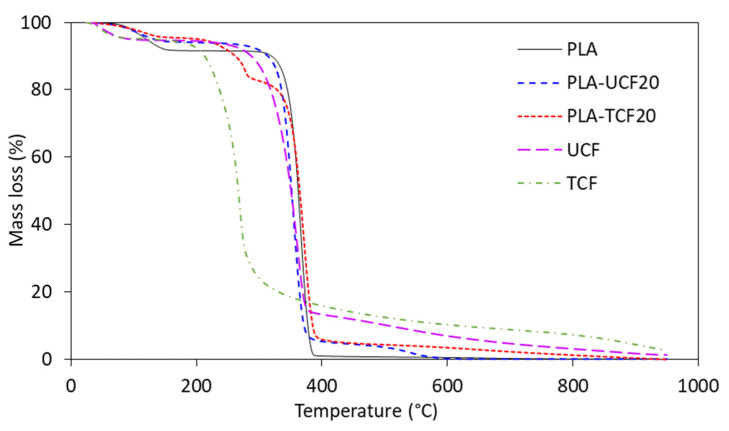
TGA curves of films and fibers with and without treatment.

**Figure 7:**
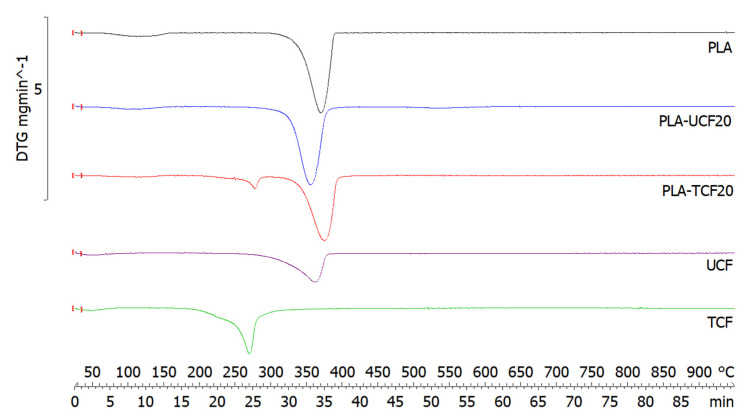
DTG curves of films and fibers with and without treatment.

Thermogravimetric analysis of PLA, untreated and treated CF and the composite films shows a multistep degradation as seen in Figure 6. The initial step (50–100 °C) can be attributed to a loss of moisture. The treated CF shows lower thermal stability and weight loss between 250–300 °C corresponding to its respective DTG peaks in Figure 7 which are mainly associated with the effect of the surfactant on the fiber [46,48]. The PLA-TCF20 shows a smaller degradation peak corresponding to the surfactant coated fibers in the matrix.

The next main degradation step for all samples occurs between 350 and 400 °C, associated with the cellulose, hemicellulose, and thermally stable compounds in the polymer matrix. The main DTG peak corresponding to the peak degradation temperature (Tmax) for PLA was 368 °C and for the PLA-CF samples it was between 354 and 372 °C, showing that there was no degradation taking place in the composites at lower temperatures. It was observed that the addition of TCF in PLA slightly increases the thermal stability of the matrix due to better reinforcement as seen in Table 3.

**Figure 8:**
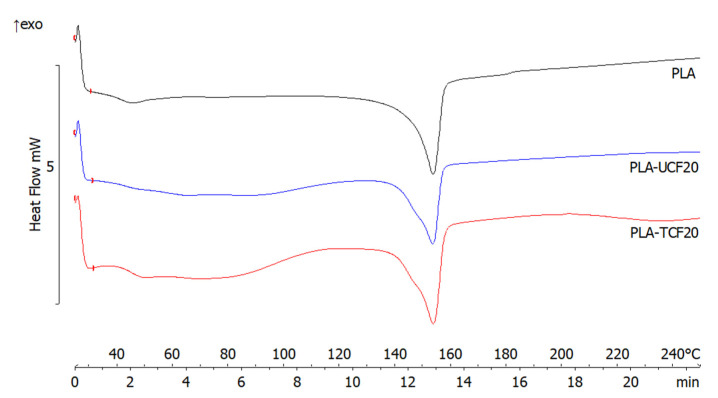
DSC curves of films with and without CF treatment.

Figure 8 shows the endothermic melting peaks for neat PLA, PLA-UCF20, and PLA-TCF20 composites. The three materials displayed similar curves and minor differences in their melting temperatures were observed, indicating that CF dispersion does not significantly affect the thermal properties of the polymer matrix. 

**Figure 9:**
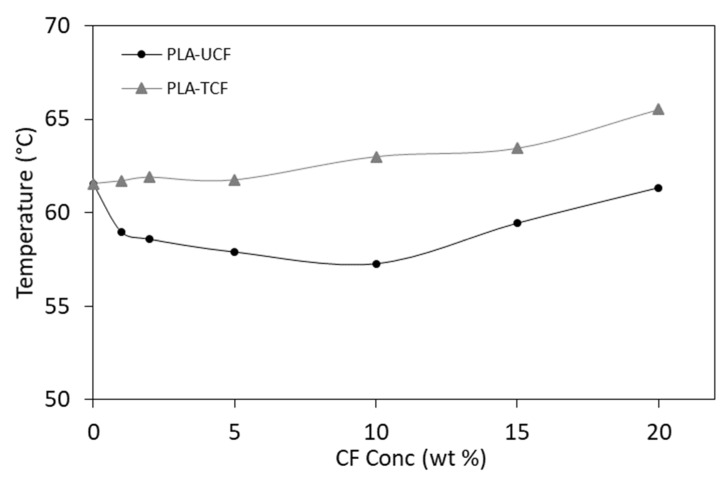
Glass transition temperature of films with varying CF concentration.

From the DMA analysis, the glass transition temperatures (Tg) of the composites were evaluated using the tan δ peak temperature. It was found that both the fiber content as well as fiber treatment had an effect on the Tg, as seen in Figure 9.

PLA composites prepared with surfactant treated fibers showed relatively higher Tg when compared to PLA composites containing untreated CF. The improvement in Tg (up to 10%) could be associated with the enhanced CF dispersion and interface compatibility due to surface modification. On the other hand, the PLA-UCF composites showed a lower Tg compared to neat PLA. This could be due to the presence of residual water in the system, which causes a degradation of PLA. Moreover, it was observed that the Tg increased with higher CF content in the PLA composites. The addition of acid phosphate ester-based surfactant has been reported to enhance dispersibility, mechanical, and stress-transfer properties of cellulose reinforced composites [49].

## 4. Conclusions

The preparation of bio-based PLA membranes incorporating micro-fibrillated cellulose fiber (CF) using a solvent casting method have been reported in this paper. The effects of CF modification, using an anionic organic phosphoric acid ester-based surfactant, on composite structure, morphology, and properties were investigated. The physical properties of the composite were significantly enhanced when prepared with treated CF. The composites containing treated CF absorbed very low amounts of water, even at 20 wt% CF loading, yet these composites were permeable to water vapour. The CF treatment resulted in a good dispersion of fibers in the PLA matrix, as seen using scanning electron microscopy, thereby reducing void formation and limiting capillary movement. However, the incorporation of treated CF did not completely block the microscopic pores in the composite samples that are needed for vapour diffusion, as seen in the vapour permeability results. The CF treatment also improved the thermal properties of the composite, increasing their glass transition temperature and peak degradation temperature. The melting point of the composites did not change before and after CF treatment. The prepared composites were breathable and showed better resistance to water. Using a relatively inexpensive treatment, smart bio-based membranes have been developed that have great potential in the building industry by increasing the hygroscopic performance as well as reducing the embodied energy of buildings. Further work is recommended considering the durability and weathering of the PLA-CF composites as well as the preparation of larger sized films and assessment of their hygroscopic performance in test cells.

## Figures and Tables

**Table 1:** Description of film samples prepared for this study.

Sample	PLA Conc. (wt%)	CF Conc. (wt%)	Surfactant Treated
PLA	100	0	-
UCF	0	100	-
TCF	0	100	Yes
PLA-UCF1	99	1	-
PLA-UCF2	98	2	-
PLA-UCF5	95	5	-
PLA-UCF10	90	10	-
PLA-UCF15	85	15	-
PLA-UCF20	80	20	-
PLA-TCF1	99	1	Yes
PLA-TCF2	98	2	Yes
PLA-TCF5	95	5	Yes
PLA-TCF10	90	10	Yes
PLA-TCF15	85	15	Yes
PLA-TCF20	80	20	Yes

**Table 2:** Vapor permeability results of the films.

Sample	WVTR (g/(h∙m²))	Thickness (mm)	Permeability P (g/(h∙m∙Pa)) × 10^−7^	Normalized WVTR (g∙m/(h∙m²)) × 10^−5^	Normalized WVTR (g∙mils/(day∙m²))
PLA	−2.21 ± 0.03	0.11 ± 0.01	−2.01 ± 0.12	−24.33 ± 1.47	−229.90 ± 13.96
PLA-UCF1	−1.26 ± 0.04	0.19 ± 0.01	−1.99 ± 0.03	−24.09 ± 0.34	−227.65 ± 3.26
PLA-UCF2	−1.32 ± 0.04	0.20 ± 0.01	−2.19 ± 0.02	−26.54 ± 0.25	−250.83 ± 2.38
PLA-UCF5	−1.70 ± 0.06	0.26 ± 0.02	−3.66 ± 0.13	−44.31 ± 1.59	−418.68 ± 15.05
PLA-UCF10	−1.98 ± 0.03	0.30 ± 0.01	−4.92 ± 0.14	−59.60 ± 1.74	−563.19 ± 16.49
PLA-UCF15	−3.16 ± 0.24	0.32 ± 0.02	−8.36 ± 0.54	−101.15 ± 6.71	−955.79 ± 60.04
PLA-UCF20	−10.28 ± 0.16	0.42 ± 0.02	−35.71 ± 1.58	−432.14 ± 19.12	−4083.21 ± 181.20
PLA-TCF1	−1.28 ± 0.03	0.19 ± 0.01	−2.01 ± 0.12	−24.40 ± 1.46	−230.60 ± 13.80
PLA-TCF2	−1.42 ± 0.04	0.19 ± 0.01	−2.24 ± 0.11	−27.16 ± 1.34	−256.61 ± 12.71
PLA-TCF5	−1.79 ± 0.06	0.25 ± 0.01	−3.71 ± 0.20	−44.87 ± 2.45	−423.97 ± 23.21
PLA-TCF10	−2.97 ± 0.13	0.20 ± 0.01	−4.91 ± 0.39	−59.46 ± 4.80	−561.85 ± 45.38
PLA-TCF15	−2.61 ± 0.10	0.26 ± 0.02	−5.61 ± 0.49	−67.92 ± 5.97	−641.81 ± 56.41
PLA-TCF20	−4.79 ± 0.15	0.25 ± 0.01	−9.92 ± 0.61	−119.97 ± 7.40	−1133.58 ± 69.90

**Table 3:** Thermal properties of films and fibers.

Sample	Melting Point (°C)	Melting Enthalpy (mJ)	Tg (°C)	Tmax (°C)	Residue (%)
PLA	153.6 ± 0.9	−128.6 ± 0.3	61.5 ± 0.5	368.1 ± 0.2	0.5 ± 0.01
PLA-UCF20	153.5 ± 0.6	−90.6 ± 0.2	61.3 ± 0.3	354.9 ± 0.1	2.4 ± 0.04
PLA-TCF20	153.7 ± 0.5	−120.2 ± 0.3	65.5 ± 0.5	372.5 ± 0.1	3.6 ± 0.03
UCF	-	-	-	361.2 ± 0.3	8.4 ± 0.04
TCF	-	-	-	269.3 ± 0.2	23.8 ± 0.12

## Data Availability

The data presented in this study are available on request from the corresponding author.
